# Do negative childhood conditions increase the risk of somatic symptoms in adolescence? – a prospective cohort study

**DOI:** 10.1186/s12889-019-7198-y

**Published:** 2019-06-26

**Authors:** Trine N. Winding, Johan H. Andersen

**Affiliations:** Department of Occupational Medicine, Danish Ramazzini Centre, University Research Clinic, Regional Hospital West Jutland, Gl. Landevej 61, 7400 Herning, Denmark

**Keywords:** Somatic symptoms, Childhood conditions, Adolescence

## Abstract

**Background:**

In order to prevent health and social problems later in life, it is important to identify childhood conditions related to the development of somatic symptoms. This prospective study expands on previous research by investigating whether negative childhood conditions are related to somatization later in life, taking other risk factors into account.

This study aims to investigate whether somatic symptoms of the participants’ parents, poor family functioning, or negative life events during childhood result in somatic symptoms in early or late adolescence.

**Methods:**

The study population includes participants from the West Jutland Cohort Study who responded to the survey on their somatic symptoms at age 15 (*n = 2963*) and/or age 18 (*n* = 2341). The study also includes additional questionnaire information about the participants’ poor family functioning, number of negative life events, and parental reports of somatic symptoms as well as register information about parental socioeconomic background. Generalized linear models for the binomial family were used and the results were presented as relative risks (RR) and risk differences (RD) with 95% confidence intervals (95%-CI).

**Results:**

Experiencing poor family functioning at age 15 showed associations with somatic symptoms at age 15 (RR 1.75, 95%-CI, 1.43–2.14 and RD 18, 95%-CI, 11–25%) and 18 (RR 1.32, 95%-CI, 1.00–1.75 and RD 7, 95%-CI, 0.2–14%). The relative risks between poor family functioning and somatic symptoms were 2.5 for the boys at age 15 and 1.71 for the girls at age 18. Having experienced two or more negative life events up to the age of 15 was associated with reporting somatic symptoms at age 15 (RR 1.73, 95%-CI, 1.31–2.28 and RD 24, 95%-CI, 11–37%). No relative risks above 1.35 were found between parents reporting somatic symptoms and participants reporting somatic symptoms at ages 15 or 18.

**Conclusions:**

An increased awareness of the association between a poor social climate in the family and somatic symptoms may help professionals in health and educational systems prevent the development of such symptoms among adolescents.

## Background

Somatic complaints are common among children and adolescents [1, 2]. Danish as well as international studies have shown that up to 40% of adolescents have experienced pain related to the neck, back, or shoulder during the previous week [2–4]. Between 20 and 30% of adolescent girls in the US experience symptoms such as headache, stomach ache, back pain and morning fatigue more than once a week [5]. Somatic complaints seem to increase steadily both in total number and severity through childhood [2, 6], and between one-third and half of children who report somatic symptoms continue to report symptoms as adults [7].

Experiencing somatic symptoms in childhood and adolescence has several negative consequences for a person’s later health and social life [8, 9]. A bidirectional association between somatic symptoms and depressive symptoms or anxiety has been documented as well as the possibility that these different symptoms are variants of the same primary disorder [8, 10]*.* Moreover, a previous Danish study found an association between somatic symptoms in adolescence and later reduced labour market participation [11]. In order to prevent these negative consequences later in life, it is relevant to identify childhood conditions related to the development of somatic symptoms.

Previous studies have shown that low family socioeconomic position is associated with higher rates of somatic symptoms among their children [5]. Furthermore, it seems that a negative parent-child relationship can affect the psychosomatic well-being of the child. A review by Eminson et al. documented that, in clinical samples, the outcome of treatment of somatic symptoms in children and adolescents was strongly linked to the family’s level of functioning [12]. Likewise, in a clinical sample of 12–17 year-olds being treated for somatic symptoms over a 12-month period, Hoffman et al. found that family functioning was strongly associated with psychosocial functioning [13]. Rhee et al. found that a lack of parental affection, involvement and control was linked to somatic complains among American adolescents in grades 7 to 12 [14]. However, an association between poor family functioning and later development of somatic symptoms has not previously been documented in a non-clinical sample of Danish adolescents.

Previous studies also demonstrate that parents’ own physical and mental health complaints are associated with increased levels of physical symptoms among their children [12]. Craig et al. found that the health anxieties of somatizing mothers are reflected in their children, who are more likely to have concerns about their own health [15], but these results are based on cross-sectional data and only include information about somatic symptoms of one of the parents. A UK population-based birth cohort study confirms and expands on these findings, documenting that childhood experience of illness in parents is an independent risk factor for later somatic symptoms [16]. Other negative life events such as parental divorce, parental illness or death are likewise found to be associated with an increased level of somatic symptoms [17, 18], but these findings do not take into account other negative childhood conditions, such as poor family function or somatic symptoms of the parents.

A review from 2011 by Schultze and Petermann identified the following family risk factors for the development of somatic symptoms: somatic symptoms of parents, psychopathology or disease of a close family member, dysfunctional family climate, traumatic experiences in childhood and insecure attachment [19]. However, only one of the studies included in the review used a prospective design, while the rest of the studies used cross-sectional or retrospective designs. As such, the review identifies a lack of prospective studies that investigate negative childhood conditions in relation to somatization and that take into account other risk factors.

In this study, we expand on previous research by including prospectively collected information about somatic symptoms from both parents and negative childhood conditions as well as information about somatic symptoms of the participant at two separate age points (early and late adolescence). By doing so, we aim to provide more precise and detailed information about the intergenerational transmission of somatic symptoms in a healthy population and to help school and health professionals detect adolescents with somatic symptoms before these symptoms develop into more persistent symptoms or serious health problems.

### Aim

This study aims to investigate whether somatic symptoms of the participants’ parents, poor family functioning, or negative life events during childhood (up to age 15) result in somatic symptoms in early adolescence (age 15) or late adolescence (age 18).

## Methods

### Design and population

Data for the present study were gathered as part of the ongoing West Jutland Cohort Study, which consists of all individuals born in 1989 and living in the county of Ringkjoebing, Denmark, in early April 2004 (*N* = 3681). Contact information for this complete regional cohort of young people was retrieved from the Central Office of Civil Registration and from public schools in the county of Ringkjoebing. The comprehensive data material contains follow-up information about the participants’ health, well-being, family, school, and work-life. Results from the West Jutland Cohort Study have previously been published in international journals [20–22]. Of the original source population, 3054 (83%) completed the initial questionnaire at age 15 (in 2004) and 2181 participants (71% of initial respondents) completed a follow-up questionnaire at age 18 (in 2007), which was conducted via email and post.

In 2004, the parents of the initial respondents completed an additional questionnaire about the parents’ self-reported somatic symptoms.

Register information about the respondents was derived from national registers in Statistics Denmark by using the personal identification number (CPR number) from the Central Office of Civil Registration. A CPR number is given to every inhabitant in Denmark at birth (or upon entry to the country for immigrants) [23]. To obtain information about family socioeconomic background, the respondents were linked to their parents or guardians using their CPR number [23].

The study population includes participants who provided questionnaire information on their somatic symptoms at age 15 (*n = 2963*) and/or age 18 (*n* = 2341).

### Ethical consideration

The study was approved by the Danish Data Protection Agency. According to Danish law (Act on Research Ethics Review of Health Research Projects), questionnaire and register-based studies require neither approval by ethical or scientific committees nor informed consent [24].

### Outcome

Questionnaire information about somatic symptoms of the participants at ages 15 and/or 18 was measured by a subscale, the symptom checklist, from the original Hopkins Symptom Checklist-90 [25, 26]. The items were chosen according to their relevance for the two age groups. At age 15, six items with a Chronbach’s alpha of 0.69 were used: whether the participants had experienced any of the following in the past 4 weeks: 1) headaches, 2) dizziness or faintness, 3) pains in heart or chest, 4) pains in lower back, 5) nausea or upset stomach, or 6) soreness of muscles. At age 18, 11 items with a Chronbach’s alpha of 0.78 were used: whether the participants had experienced any of the following in the past 4 weeks 1) headaches, 2) dizziness or faintness, 3) pains in heart or chest, 4) nausea or upset stomach, 5) soreness of muscles, 6) trouble breathing, 7) hot or cold spells, 8) numbness or tingling in parts of the body, 9) a lump in the throat, 10) feeling weak in parts of the body, or 11) heavy feeling in arms or legs. The response categories (not at all, a little, moderately, quite a bit, extremely bothered) were generated into scales ranging from 0 to 24 (age 15) and 0–44 (age 18) and dichotomized at the 75th percentile into low/high somatization, with a cut-off at ≥5 and ≥ 8, respectively. We made the pragmatic decision to dichotomize at the 75th percentile so that we could explore contrasts in the material.

### Exposures

Questionnaire information about the parents’ somatic symptoms was measured when the participants were 15 years of age using five items from the subscale described above [25] whether the parents had experienced any of the following in the past 4 weeks: 1) headaches, 2) pains in lower back, 3) soreness of muscles, 4) numbness or tingling in parts of the body, or 5) feeling weak in parts of the body. Information was gathered about both the mother and father. The Chronbach’s alphas of the somatic symptoms scale for the mother and father were 0.76 and 0.71 respectively. The five response categories were generated into scales ranging from 0 to 20 and dichotomized at the 75th percentile into low/high somatization, with a cut-off at ≥3, for both mothers and fathers.

The social climate in the family was measured by questionnaire when the participants were 15 years of age using the General Functioning Scale, which is one of seven scales from the Family Assessment Device [27]. The General Functioning Scale is made up of 12 items with an alpha of 0.85 and measures the overall health/pathology of the family, where low scores indicate healthier functioning than higher scores. The General Functioning Scale was dichotomized at the 75th percentile into good/poor family functioning, with a cut-off at 2.08 [27].

Negative life events up to age 15 were assessed using six items, partly from a scale developed by Newcomb, Huba, and Bentler [28] and partly from The Social Stress Indicator developed by Turner, Wheaton, and Lloyd [29]. The six questionnaire items asked participants at age 15 about negative life events such as divorce of parents, parental drug or alcohol abuse, disease or death in the immediate family, violent events or physical abuse. The number of negative life events was dichotomized into ‘0–1 events’ and ‘2 or more events’.

Socioeconomic background was defined according to highest attained education in the household and household income in the year 2003. Based on the source population (*N* = 3681), yearly household income was divided into tertiles corresponding to lowest (< 61,931 EUR), middle (61,931–80,738 EUR), and highest (> 80,738 EUR) income category [30]. The highest attained education in the household was recoded into three categories: < 10 years, 10–12 years, > 12 years [31]. If the participants’ parents were divorced, as was the case for 28% of the participants in 2004, information was obtained from the household at which the participants’ address was listed.

Information about gender was based on register information [23].

### Statistical analysis

A correlation analysis between all exposure variables was performed initially and showed a correlation of *r* = 0.3 between household income and parental highest education. All other correlations were below r = 0.16. Potential interactions between somatization of both the mother and the father and between negative life events and family functioning in relation to development of somatic symptoms in early or late adolescence were tested, but none of the interaction terms contributed to the models.

The exposure variables were initially included in the models as continuous or categorical variables. A sensitivity analysis was then performed to determine whether the main results were affected when then cut-off points were changed. Since this was not the case, the exposure variables were dichotomized to improve interpretability of the results. The prevalence’s of all outcome and exposure variables were presented separately for girls and boys and chi^2^-tests were performed to test for gender differences (Table 1).

**Table 1:** Characteristics of the participants (n,%), *N* = 3223

	All		Girls (*n* = 1622)		Boys (*n* = 1601)		*P*-value
	n	%	n	%	n	%	
Somatic symptoms (age 15)	2963		1493		1470		< 0.001
few	2103	71	916	61	1187	81	
many	860	29	577	39	283	19	
Somatic symptoms (age 18)	2341		1262		1079		< 0.001
few	1748	75	837	66	911	84	
many	593	25	425	34	168	16	
Somatic symptoms mother (age 15)	1760		885		875		0.985
few	1167	66	587	66	580	66	
many	593	34	298	34	295	34	
Somatic symptoms father (age 15)	1502		746		756		0.282
few	1070	71	522	70	548	72	
many	432	29	224	30	208	28	
Family functioning (age 15)	2880		1451		1429		0.691
good	2137	74	1072	74	1065	75	
poor	743	26	379	26	364	25	
Number of negative life events, up to age 15	2959		1483		1476		0.082
0–1	2566	87	1270	86	1296	88	
2 or more	393	13	213	14	180	12	
Parental household income (age 14)	3221		1620		1601		0.219
high	1139	35	563	35	576	36	
medium	1109	34	545	34	564	35	
low	973	30	512	32	461	29	
Highest education in the household (age 14)	3166		1591		1575		0.082
high	1136	36	542	34	594	38	
medium	1635	52	839	53	796	51	
low	395	12	210	13	185	12	

Generalized linear models for the binomial family were used to analyze the association between parental somatic symptoms, family functioning or number of negative life events up to the age of 15, and somatic symptoms of the child at ages 15 (Table 2) or 18 (Table 3). The results are presented as relative risks (RR) and risk differences (RD) with 95% confidence intervals (95%-CI). Crude estimates, estimates adjusted for household income and highest education in the household (partly adjusted), and estimates adjusted for socioeconomic variables and all other independent variables (fully adjusted) were presented (Tables 2 and 3). Gender specific analyses were performed, and substantial differences in risk estimates between the two genders are mentioned in the result section. Level of significance was set at *p* = 0.05. All analyses were carried out in STATA statistical package (V.15.0; State, College Station, TX).

**Table 2:** Childhood conditions related to somatic symptoms at age 15, RR and RD with 95%-CI, *N* = 2963

	Crude			Partly adjusted^b^			Fully adjusted^c^		
	Prevalence^a^ (%)	RR (95%-CI)	RD (95%-CI) (%)	Prevalence^a^ (%)	RR (95%-CI)	RD (95%-CI) (%)	Prevalence^a^ (%)	RR (95%-CI)	RD (95%-CI) (%)
Somatic symptoms mother age 15, *n* = 1760									
few	25	ref	ref	24	ref	ref	16	ref	ref
many	33	1.28 (1.10;1.49)	7 (3;12)	31	1.29 (1.11;1.51)	7 (3;12)	23	1.25 (1.03;1.52)	8 (2;13)
Somatic symptoms father age 15, *n* = 1502									
few	27	ref	ref	24	ref	ref	16	ref	ref
many	30	1.14 (0.95;1.35)	4 (−1;9)	27	1.12 (0.94;1.33)	3 (−2;8)	17	1.02 (0.83;1.26)	1 (−5;7)
Family functioning age 15, *n* = 2880									
good	25	ref	ref	23	ref	ref	16	ref	ref
poor	42	1.69 (1.50;1.89)	17 (13;21)	41	1.69 (1.50;1.90)	17 (13;21)	34	1.75 (1.43;2.14)	18 (11;25)
Number of negative life events up to age 15, *n* = 2959									
0–1	27	ref	ref	26	ref	ref	16	ref	ref
2 or more	40	1.50 (1.31;1.72)	13 (8;19)	40	1.51 (1.31;1.75)	14 (8;19)	40	1.73 (1.31;2.28)	24 (11;37)

^a^Prevalence of somatic symptoms at age 15 in relation to the different exposure categories

^b^Partly adjusted: adjusted for parental household income and highest education in the family

^c^Fully adjusted: adjusted for parental household income, highest education in the family, and all other childhood conditions, *n* = 1073

**Table 3:** Childhood conditions related to somatic symptoms at age 18, RR and RD with 95%-CI, *N* = 2341

	Crude			Partly adjusted^b^			Fully adjusted^c^		
	Prevalence^a^ (%)	RR (95%-CI)	RD (95%-CI) (%)	Prevalence^a^ (%)	RR (95%-CI)	RD (95%-CI) (%)	Prevalence^a^ (%)	RR (95%-CI)	RD (95%-CI) (%)
Somatic symptoms mother									
age 15, *n* = 1760									
few	22	ref	ref	16	ref	ref	14	ref	ref
many	29	1.33 (1.10;1.61)	7 (2;12)	23	1.28 (1.06;1.55)	6 (2;11)	16	1.25 (0.97;1.61)	5 (−0.6;11)
Somatic symptoms father									
age 15, *n* = 1502									
few	22	ref	ref	19	ref	ref	14	ref	ref
many	29	1.32 (1.07;1.62)	7 (1;13)	25	1.29 (1.04;1.59)	6 (0.4;12)	22	1.35 (1.04;1.74)	7 (0.9;14)
Family functioning									
age 15, *n* = 2880									
good	23	ref	ref	20	ref	ref	14	ref	ref
poor	31	1.35 (1.15;1.58)	8 (3;13)	27	1.30 (1.10;1.53)	7 (2;12)	22	1.32 (1.00;1.75)	7 (0.2;14)
Number of negative life events									
up to age 15, *n* = 2959									
0–1	23	ref	ref	21	ref	ref	14	ref	ref
2 or more	37	1.61 (1.33;1.94)	14 (7;21)	34	1.54 (1.26;1.89)	14 (6;21)	23	1.25 (0.79;1.99)	9 (−5;22)

^a^Prevalence of somatic symptoms at age 15 in relation to the different exposure categories

^b^Partly adjusted: adjusted for parental household income and highest education in the family

^c^Fully adjusted: adjusted for parental household income, highest education in the family, and all other childhood conditions, *n* = 895

## Results

As shown in Table 1, a higher proportion of girls reported many somatic symptoms at ages 15 and 18 (39 and 34% respectively) compared with boys (19 and 16% respectively). Approximately 13% of all participants had experienced two or more negative life events at age 15; this applied slightly more to girls than boys (14% vs. 12%), but no significant gender differences were found in relation to the distribution of any of the independent variables.

The associations between the socioeconomic variables and somatic symptoms at ages 15 or 18 were not statistically significant, including the association between parental education and somatic symptoms at age 18 (fully adjusted: RR 1.19, 95%-CI, 0.74–1.89 and RD 5, 95%-CI, − 8-17%).

As shown in Table 2, the childhood conditions showing statistically significant associations with somatic symptoms of all the participants at age 15 were poor family functioning (fully adjusted: RR 1.75, 95%-CI, 1.43–2.14 and RD 18, 95%-CI, 11–25%) and having experienced two or more negative life events (fully adjusted: RR 1.73, 95%-CI, 1.31–2.28 and RD 24, 95%-CI, 11–37%). For boys experiencing poor family function, the risk of developing somatic symptoms was approximately 2.5 times higher than boys from well-functioning families, and 33% more boys from poor-functioning families reported somatic symptoms than boys from well-functioning families (results not shown). Somatic symptoms of the mother showed an association with self-reported somatic symptoms at age 15 for the whole sample (fully adjusted: RR 1.25, 95%-CI, 1.03–1.52 and RD 8, 95%-CI, 2–13%) and in girls (fully adjusted: RR 1.45, 95%-CI, 1.21–1.74 and RD 15, 95%-CI, 6–24%).

As shown in Table 3, the childhood conditions showing significant associations with somatic symptoms of all participants at age 18 were somatic symptoms of the father (fully adjusted: RR 1.35, 95%-CI, 1.04–1.74 and RD 7, 95%-CI, 0.9–14%) and poor family functioning (fully adjusted: RR 1.32, 95%-CI, 1.00–1.75 and RD 7, 95%-CI, 0.2–14%). Family functioning showed a significant association with somatic symptoms at age 18 among the girls (fully adjusted: RR 1.71, 95%-CI, 1.32–2.21 and RD 14, 95%-CI, 3–25%).

## Discussion

In this study, we found statistically significant associations between experiencing poor family functioning and reporting somatic symptoms at ages 15 or 18, when adjusted for other childhood risk factors. The relative risks were 2.5 for the boys at age 15 and 1.71 for the girls at age 18. Negative life events up to the age of 15 showed a significant association with reporting somatic symptoms at age 15, but the association was not significant at age 18. No relative risks above 1.35 were found between parents reporting somatic symptoms and the participants reporting somatic symptoms at ages 15 or 18.

To our knowledge, this is the first prospective study that examines the associations between several negative childhood conditions, including somatic symptoms of the parents and reporting of somatic symptoms in adolescence in a population-based sample.

Poor family functioning showed a significant association with somatic symptoms at age 15 and 18. These findings are in line with the results of previous studies [14, 32, 33]. In a Swedish cohort, Landstedt et al. found that poor parental and peer relationships at age 16 continued to be associated with functional somatic health symptoms for up to 26 years [32], and a cross-sectional study by Hart et al. found that family conflict was associated with clinically significant somatic complaints reported by elementary school children [33]. However, the latter study included data from a predominantly African American urban study population, which may limit the ability to generalize the findings to a Danish population. The results of this study show that social workers and teachers in contact with adolescents should be aware of the adolescents’ family situation if they wish to prevent the development of somatic symptoms.

In line with earlier findings, we found that somatic symptoms of the parents was associated with somatic symptoms of the children [19, 34]. However, at both age 15 and 18, the relative risks were not above 1.35. A study by Janssens et al. found that 11–16 year-olds whose parents reported high rates of functional somatic symptoms were more than four times as likely to report persistent functional somatic symptoms [34]. Our study did not find such a strong association, which is perhaps because we did not measure persistent symptoms and thus most likely measured less chronic conditions. Another study by Craig et al. found that children of somatizing mothers were more likely to experience emotional or behavioral problems, have greater concerns about their own health, and have higher consultation rates for functional somatic symptoms [15]. In our study, we used self-reported information about somatic symptoms of both the mother and the father. To our knowledge, such detailed cross-generational information about somatic symptoms has not yet been reported. It seems that having a somatizing mother increases the risk of girls having somatic symptoms at age 15. Somatic symptoms of the father were associated with somatic symptoms of their children at age 18 and the estimates where relatively robust to adjustments.

Earlier prospective studies have shown that negative life events in childhood are associated with somatic symptoms in adolescence in both genders [16–18, 35] and in girls only [36], but this has not previously been documented in a Danish population of young people. In our study, the association between negative life events and somatic symptoms was significant at age 15, whereas the association was not significant at age 18. It is possible that the association would have been significant if some of the questions regarded negative life events of a more serious nature. For example, as mentioned above, one of the questions regarded divorce of the parents, which does not identify an uncommon life event in Denmark.

In this study, the outcome was measured at the two ages 15 and 18. We consider these ages relevant for measuring somatic symptoms in early and late adolescence, because they represent particularly sensitive life periods [37].

This study has several strengths, including its longitudinal design and relatively large sample size. Moreover, the study examines a population-based sample, which increases the generalizability of its results. Another strength is that data about somatic symptoms were available from participants at both ages 15 and/or 18 and from both of their parents, which allowed us to examine the course of somatic symptoms throughout adolescence and to differentiate between somatic symptoms of the father and the mother. Finally, the use of both register and questionnaire data minimizes the risk of common method variance and thereby the risk of bias [38].

Some limitations of the study also have to be taken into account. Despite the large sample size, the group of participants with complete information about outcome and all exposures was only *n* = 1073 (36%) and *n* = 895 (38%) at age 15 and 18 respectively. This was primarily due to missing information about father’s somatic symptoms. A supplementary analysis was performed to investigate how the estimates changed if only those who had answered information about somatic symptoms at both age points were included. The most significant change in estimate was seen in relation to negative life events and somatic symptoms at age 15, where the complete case analysis increased the relative risk by 0.24 and the risk difference by 7%. All other estimates showed inconsiderable changes.

Another limitation was the use of different items when generating the somatic symptoms scales at age 15 and 18. Although the two scales contained five of the same items, it is a limitation of the study that the number of items, and thus symptoms asked about, was different at the two age collection points. Supplementary analyses showed that when using only the five identical items both at age 15 and 18 only minor changes were seen. The biggest change in estimate was a decrease of 0.25 in relative risk.

It would have been possible to use another instrument to measure negative life events, such as the ACE questionnaire, though many of the items in the ACE questionnaire are similar to those used in this study [39].

Since information about family functioning and somatic symptoms at age 15 was collected at the same time point, it is possible that negative affectivity could have played a role. In other words, it is possible that poor family functioning influences the way an individual perceives and reports his/her current symptoms, meaning that those adolescents in our study who reported poor family functioning could have automatically reported more somatic symptoms. This problem could potentially have led to differential misclassification and an overestimation of the association between family functioning and somatic symptoms. However, since this study shows associations between family functioning at age 15 and the reporting of somatic symptoms both at age 15 and 18, this potential bias is most likely limited. Another limitation of the study is that it was not possible to adjust for chronic illness among the participants. It is possible that the reported symptoms were caused by some kind of chronic illness. However, chronic illness would most likely be associated with the outcome and not the exposures and would therefore not lead to differential misclassification [40].

In prospective cohort studies, there will always be some selection based on participation, but, when comparing parental income and educational level of the source population and the study population, only small differences were seen. The prevalence of families from the lowest percentile decreased from 33 to 30%, and families with less than 10 years of education decreased from 14 to 12.5%. Information about somatic symptoms was only available for approximately half of the fathers. If the father’s participation was related to both his own somatization and the degree of somatic symptoms of the child, this could potentially have biased the risk estimates. When comparing the prevalence of somatic symptoms among those children whose fathers responded and those children whose fathers did not respond, we only found minor differences in the two groups corresponding to 28 and 30%, respectively.

A previous study on the Vestliv Cohort found that selection due to participation does not necessarily significantly influence the risk estimates measured [41]. Therefore, the potential selection bias is most likely minor.

## Conclusions

Our findings indicate that early negative childhood conditions, especially poor family functioning and negative life events, are associated with somatic symptoms at ages 15 and/or 18. This emphasizes the importance of professionals in health and educational system paying more attention to emotional conditions in the family, family dynamic and negative events in order to prevent adolescents from developing somatizing tendencies.

## Data Availability

The datasets used and/or analysed during the current study are available from the corresponding author on reasonable request.

## Abbreviations

CI: Confidence interval
RD: Risk difference
RR: Relative risk
